# Opportunistic Pathogens Mycobacterium Avium Complex (MAC) and *Legionella* spp. Colonise Model Shower

**DOI:** 10.3390/pathogens4030590

**Published:** 2015-07-24

**Authors:** Harriet Whiley, Steven Giglio, Richard Bentham

**Affiliations:** 1 School of the Environment, Health and the Environment, Flinders University, GPO BOX 2100, Adelaide 5001, Australia; E-Mail: richardhbentham@gmail.com; 2 Healthscope Pathology SA, 1 Goodwood Rd, Wayville 5034, South Australia; E-Mail: Steven.Giglio@healthscope.com.au

**Keywords:** Mycobacterium avium complex (MAC), non-tuberculous mycobacterium (NTM), *Legionella* spp., *Legionella pneumophila*, potable water, showers, model, biofilm

## Abstract

*Legionella* spp. and Mycobacterium avium complex (MAC) are opportunistic pathogens of public health concern. Hot water systems, including showers, have been identified as a potential source of infection. This paper describes the colonization of *Legionella* and MAC on the flexible tubing within a model potable shower system, utilizing thermostatic mixing and a flexible shower head. A MAC qPCR method of enumeration was also developed. MAC and *Legionella* spp. were detected within the biofilm at maximum concentrations of 7.0 × 10^4^ and 2.0 × 10^3^ copies/cm^2^ PVC tubing respectively. No significant changes were observed between sample of the flexible shower tubing that dried between uses and those that remained filled with water. This suggested the “unhooking” showerheads and allowing them to dry is not an effective method to reduce the risk of *Legionella* or MAC colonisation.

## 1. Introduction

Contaminated shower systems are a major public health concern as they may result in constant exposure to potentially harmful microorganisms [1]. Showers provide an ideal niche for bacterial biofilm formation, which enables both pathogenic and opportunistic microorganisms to accumulate in large numbers. These microorganisms are then dispersed into the passing water and aerosolised by the showerhead, providing means for inhalation deep into the respiratory tract [2]. Mycobacterium avium complex (MAC) and *Legionella* spp. are opportunist human pathogens, that have been associated with contaminated shower systems as a source of infection [3,4].

MAC causes a wide spectrum of disease including: fibrocavitary lung disease [5], fibronodular bronchiectasis [6], pulmonary nodules simulating lung cancer [7], hypersensitivity pneumonitis [8], cutaneous skin [9] and soft tissue infection particularly in post-surgery patients [10], cervical lymphadenitis in children [11], gastrointestinal tract and disseminated infection in immune compromised patients [12], and debatably Crohns disease [13]. In most countries MAC is not a notifiable disease and its wide range of clinical manifestations makes it is difficult to accurately estimate disease prevalence; however, it is generally accepted that the incidence of MAC infection is increasing [14,15,16].

One hypothesis is that this increased prevalence is related to the change in our hygiene habits from bathing to showering [17]. This was further supported by a study in Japan which investigated the distribution of MAC in domestic households. MAC was only recovered from bathrooms but not from other sites of residences and the prevalence of detection in the bathrooms of patients with pulmonary MAC was significantly higher than that in healthy volunteers’ bathrooms [18].

*Legionella* spp. is the causative pathogen of Legionellosis which includes Legionnaires’ disease, a *Legionella* derived pneumonic infection, and Pontiac fever, an acute febrile illness [19]. The exact incidence of Legionellosis worldwide is unknown due to differences in diagnosis and reporting methods. In the United States it is estimated that 8000 to 18,000 people contract Legionnaires’ disease annually, with surveillance data from the Centres for Disease Control and Prevention (CDC) indicating that less than 10% of estimated cases are reported to local and state health officials [20]. Studies investigating nosocomial outbreaks of Legionnaires disease have identified contamination of hospital water distribution systems [21,22] and specifically shower heads as a likely source of infection [23].

The aim of this study was to develop a model potable warm water system to simulate a shower utilizing thermostatic mixing and a flexible shower head, as typically found in hospitals aged care facilities and domestic households.

## 2. Experimental Model and Methods

**Figure 1:**
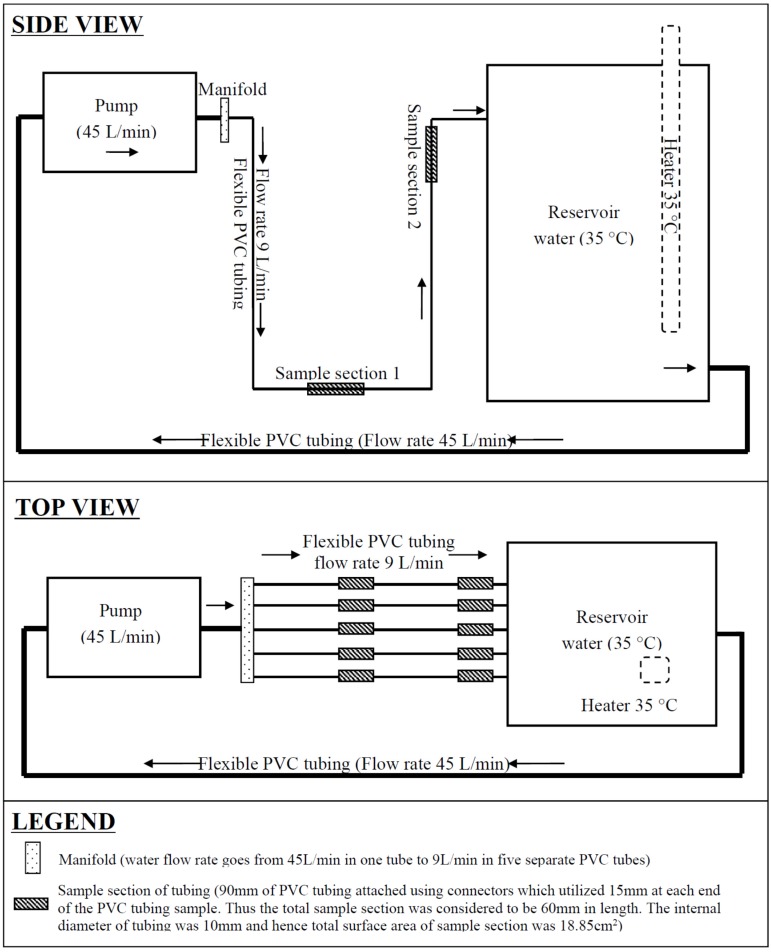
Diagram of model potable warm water shower system.

The model (Figure 1) was constructed so that two sections of each PVC flexible tubing replicate (with an internal diameter of 10 mm as commonly used in shower heads) could be destructively sampled. Sample 1 was positioned in the “u bend” of the flexible tubing and remained filled with water between uses; whereas, sample 2 was positioned near the top section of tubing which drained dry between uses. This was done to represent a flexible shower head left on its hook and to determine if allowing flexible shower heads to drain in-between uses has an effect on the risk for *Legionella* or MAC colonisation.

The novelty of our approach is the qPCR quantification and study of temporal variations of these opportunist pathogens in operating showers systems. This includes showers that have been allowed to drain which have been suggested to be less likely to be colonised than those with residual water in them. This study has demonstrated that there is no significant change to population densities in frequently used hoses whether left to drain or “hung up”. To our knowledge this has not been previously reported.

Potable water was added to the reservoir and the temperature of the water was maintained at 35 °C to represent thermostatic mixing. The flow rate in each PVC tubing was 9 L/min as considered the maximum flow rate for water saving shower heads. Water was pumped for 3 min a day for 5 days on, two days off, for 6 weeks to allow the biofilm to establish. Once the system had established the detachable sampling sections of tubing were replaced with clean tubing and the water continued to be pumped for 3 min a day for 5 days on, two days off. This temporal water usage pattern was selected as it represents the non-continuous usage that is likely for hospital showers. Swab samples were taken from the pipe surface not from the bathing medium to characterise the established biofilm population. Sample sections of tubing were removed at weekly intervals to investigate the time that it took for biofilm to establish (each of the five tubing replicates represented another week of biofilm formation). This sampling timeline was repeated three times. On test days the sections of tubing were removed prior to flushing for that day. Once a sample was removed the tubing was sliced in half and swabbed with a sterile cotton tip. The two halves of tubing and the sterile swab were submerged in 45 mL of sterile water and mixed by inversion for 30 s, vortexed for 60 s and sonicated at 46 kHz for 3 min. The tubing and cotton tips were then removed with sterile tweezers.

qPCR was chosen as the enumeration method over culture, as culture is notoriously inaccurate and can return false negative results [24]. Culture is especially problematic for MAC, with very poor recoveries and long incubation periods that require molecular confirmation of presumptive culture results [25,26]. DNA was extracted from 40 mL of each water sample using the BIO-RAD Aquadien^TM^ Kit following manufacturer’s instructions (Bio-Rad Laboratories, Inc., Hercules, CA, USA). *Legionella* spp. were enumerated using a previously described qPCR method, using primers JFP 5'-AGGGTTGATAGGTTAAGAGC-3' and JRP 5'-CCAACAGCTAGTTGACATCG-3' [27]. MAC qPCR was developed using previously described primers MACF (5'-CCCTGAGACAACACTCGGTC-3') and MACR (5'-ATTACACATTTCGATGAACGC-3') [28].The limit of detection of the MAC assay was determined using a series on 1 in 10 dilutions (5 × 10^0^–5 × 10^9^ copies/reaction) of purified PCR product using the Corbett Research liquid handling system. The limit of detection was 50 copies/reaction which equated to a limit of detection of 96 copies/cm^2^.

The optimised 25 µL reaction volume contained 1 × PCR buffer (Invitrogen), 2.5 mM MgCl_2_ (Invitrogen), 2.5 mM SYTO9 fluorescent dye (Invitrogen), 0.2 mM deoxynucleoside triphosphate mix (Invitrogen), 1 U platinum Taq DNA polymerase (Invitrogen), 0.3 µM forward primer, 0.3 µM reverse primer and 5 µL of template DNA. The cycling conditions included an initial hold at 95 °C for 5 min, followed by 45 cycles consisting of 94 °C for 10 s, 60 °C for 20 s, and 72 °C for 20 s.

All qPCR reactions were carried out in a RotorGene 3000 (Corbett Research, Sydney, Australia) with data acquisition at 72 °C on the 6-carboxyfluorescein channel (excitation at 470 nm, detection at 510 nm) at a gain of 5. Melt curve data was also acquired on this channel at gains of 2 and 5 using a ramping rate of 1 °C/60 s from 75 °C to 95 °C. For each reaction the melt curve was analysed and a positive *Legionella* spp. and MAC was confirmed with a melting temperature (T*_m_*) of 88 ± 1 °C and 85 ± 1 °C respectively. Quantification of confirmed amplicons for both MAC and *Legionella* was performed using the standard curves and linear regression equations generated by the Rotorgene software.

## 3. Results and Discussion

**Figure 2:**
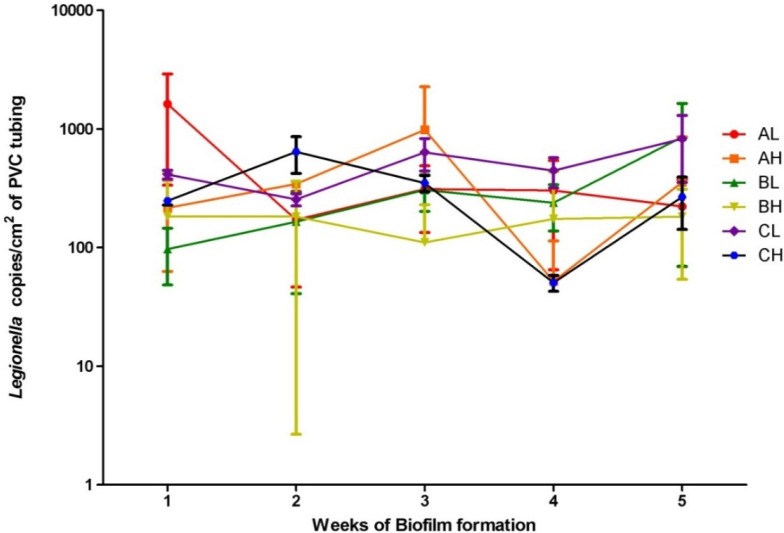
*Legionella* spp. copies/cm^2^ of PVC tubing and standard deviations (n = 3) of sample 1 (L) and sample 2 (H) sample sections from the three sampling replicates (A, B and C) from the model potable water system over 5 weeks of biofilm formation displayed on a logarithmic scale.

**Figure 3:**
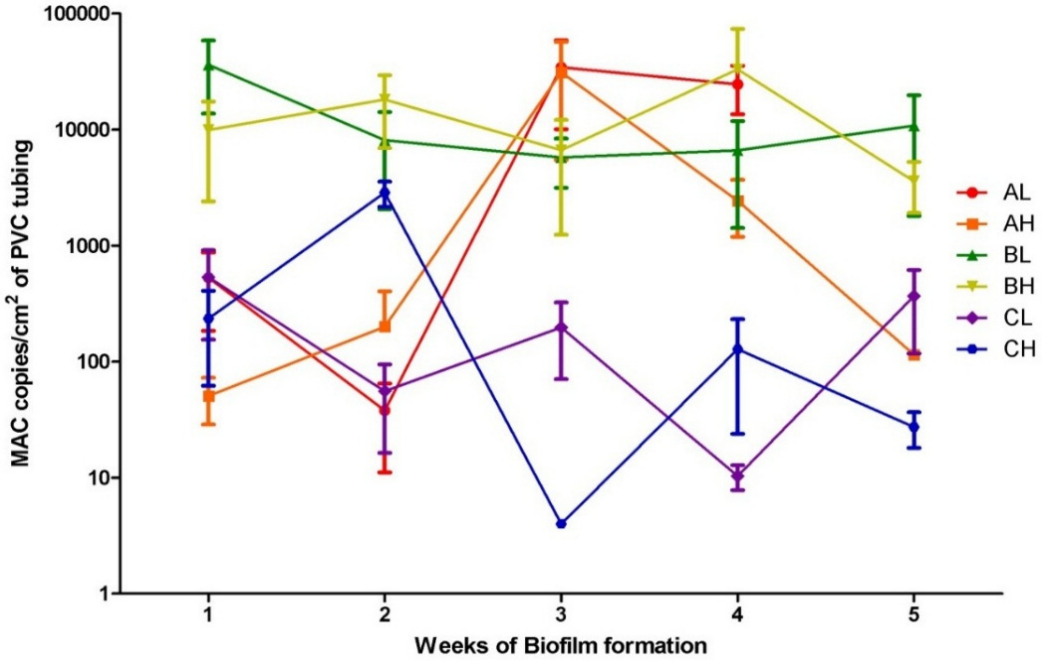
Mycobacterium avium complex (MAC) copies/cm^2^ of PVC tubing and standard deviations (n = 3) of sample 1 (L) and sample 2 (H) sample sections from the three sampling replicates (A, B and C) from the model potable water system over 5 weeks of biofilm formation displayed on a logarithmic scale.

The South Australian potable water added to the model system was not spiked with positive cultures; however, MAC and *Legionella* spp. were detected at maximum concentrations of 7.0 × 10^4^ and 2.0 × 10^3^ copies/cm^2^ of PVC tubing. Although qPCR does not differentiate between viable and killed cells [29], the ability to colonise the biofilm on PVC tubing indicates viability and suggests their presence in South Australian potable supply. Statistical analyses of results were conducted using Graph Pad^TM^ prism 5.0 (Graph Pad software Inc., La Jolla, CA, USA). Kolmogorov-Smirnov normality test of MAC and *Legionella* copies/cm^2^ determined that all samples were not normally distributed. The transformed log_10_ of MAC and *Legionella* copies/cm^2^ data was then tested again using the Kolmogorov-Smirnov normality test and were found to be normally distributed. The log_10_ of results were then used for all further statistical analyses and are presented in Figure 2 and Figure 3. A one way ANOVA of the log_10_ of the MAC and *Legionella* copies/cm^2^ was then conducted to determine if the means of each time point were significantly different to the 95% confidence level. The resulting P values were 0.0032 and 0.0419 respectively (*p* < 0.05) indicating that they were significantly different and the concentration of biofilm changed over time, although no trends were observed. For each of the three replicates, an unpaired T-test of MAC and *Legionella* numbers at sample 1 (low sample) and sample 2 (high sample) over each time period was conducted to demonstrate that the mean log_10_ MAC (*p* = 0.5646, 0.8825, 0.8967) and *Legionella* (*p* = 0.3150, 0.0541, 0.1704) copies/cm^2^ detected at sample 1 and sample 2 of the same time point were not significantly different. This suggests that the *Legionella* and MAC were not affected by desiccation between flushing and that the once a day flushing did not provide sufficient shearing force to remove biofilm or prevent its formation. Thus “unhooking” flexible shower heads and allowing them to dry does not reduce the amount of *Legionella* formation. 

## 4. Conclusions

This model shower system demonstrated the potential of MAC and *Legionella* spp. to colonise the plastic tubing in flexible shower heads and that temporal water usage had no effect on their colonisation. Also for each sampling time point, there was no significant difference in the concentration of *Legionella* or MAC sampled from a tubing section that was allowed to dry out and a section that was filled with water in between usages. This suggests that allowing shower tubing to dry between usages will not reduce the risk for *Legionella* or MAC colonisation. This information is useful for future consideration of environmental factors influencing the contamination of shower systems in aged care facilities hospitals and domestic households using flexible shower heads.
